# Supplementary material for: Multisensory input modulates memory-guided spatial navigation in humans
Source: Commun Biol. 2023 Nov 14;6:1167. doi: 10.1038/s42003-023-05522-6 (PMC10646091; doi:10.1038/s42003-023-05522-6)
Supplement: Supplementary file 2 — Supplementary material [file 42003_2023_5522_MOESM2_ESM.pdf]

Supplementary Information

Supplementary table 1

Supplementary table 1. Spatial configuration of the six presented blocks.

Block	Origin angle	Target angle	Angle between origin and target	Target distance from center (meters/virtual meters)
1	0	315	-45	2.63
2	300	165	-135	2.77
3	240	15	135	2.52
4	180	225	45	2.03
5	120	75	-45	2.68
6	60	195	135	2.75

Supplementary method 1

Trial order of each block:

Disorientation task – 1st learning trial – disorientation task – 2nd learning trial – disorientation task – 3rd learning trial

Disorientation task – 1st probe trial – disorientation task – 2nd probe trial – disorientation task – 3rd probe trial – disorientation task – 4th probe trial

Supplementary table 2

Supplementary table 2. Table of results for probe trials.
Data presented as mean ± s.e.m and 95%-confidence interval. In case main analysis revealed an interaction effect, post-hoc tests were performed with the Holm-Bonferroni-correction.

	Probe trials				
	Stationary		Mobile		
	**MTLR** (n = 10)	**Control** (n = 20)	**MTLR** (n = 10)	**Control** (n = 20)	
**Spatial memory**					p-value
Memory score in percent	72.4 ± 5.4 a<br>60.1 – 84.6	87.6 ± 2.1 b<br>83.3 – 91.9	89.5 ± 1.8 c<br>85.4 – 93.5	94.6 ± 1.0 d<br>93.1 – 96.0	G*S: 0.031<br>a*b < 0.001<br>a*c < 0.001<br>b*d = 0.031<br>c*d = 0.251
**Spatial precision**					
Scatter as distance in virtual units	0.23 ± 0.03 a<br>0.16 – 0.30	0.12 ± 0.01 b<br>0.10 – 0.15	0.09 ± 0.01 c<br>0.07 – 0.12	0.06 ± 0.00 d<br>0.05 – 0.07	G*S: 0.003<br>a*b < 0.001<br>a*c < 0.001<br>b*d = 0.002<br>c*d = 0.194
**Navigation efficiency**					
Latency to final location in seconds	25.8 ± 2.7<br>19.8 – 31.8	27.5 ± 1.8<br>23.6 – 31.4	16.4 ± 1.6<br>12.8 – 20.1	18.0 ± 1.4<br>15.1 – 20.9	G*S: 0.955<br>S: < 0.001<br>G: 0.423
Path error to final location in percent	278.8 ± 55.1<br>154.2 – 403.3	251.2 ± 31.9<br>184.4 – 318.0	124.3 ± 28.1<br>60.8 – 187.9	116.2 ± 15.5<br>83.7 – 148.7	G*S: 0.682<br>S: < 0.001<br>G: 0.611
Surface coverage in percent	24.9 ± 2.8<br>18.5 – 31.4	22.9 ± 1.6<br>19.6 – 26.1	16.2 ± 1.9<br>11.9 – 20.5	18.6 ± 1.7<br>15.1 – 22.2	G*S: 0.128<br>S: < 0.001<br>G: 0.929
**Navigation strategies**					
Search accuracy/ avg. distance to final location in virtual units	0.33 ± 0.02<br>0.28 – 0.39	0.30 ± 0.01<br>0.27 – 0.33	0.28 ± 0.02<br>0.23 – 0.32	0.25 ± 0.02<br>0.21 – 0.28	G*S: 0.810<br>S: < 0.001<br>G: 0.212
Angular velocity/ idPhi (Integrated over the initial 5 seconds)	0.0036 ± 0.0005<br>0.0024 – 0.0048	0.0041 ± 0.0003<br>0.0034 – 0.0048	0.0080 ± 0.0004<br>0.0071 – 0.0088	0.0107 ± 0.0005<br>0.0096 – 0.0117	G*S: 0.024<br>a*b = 0.477<br>a*c < 0.001<br>b*d < 0.001<br>c*d < 0.001
Path replication/ distance between paths in virtual units	0.30 ± 0.01<br>0.27 – 0.32	0.26 ± 0.01<br>0.25 – 0.28	0.23 ± 0.02<br>0.19 – 0.26	0.26 ± 0.01<br>0.24 – 0.28	G*S: 0.002<br>a*b = 0.063<br>a*c = 0.002<br>b*d = 0.759<br>c*d = 0.079

Supplementary table 3

Supplementary table 3. Table of results for learning trials.
Data presented as mean ± s.e.m and 95%-confidence interval. In case main analysis revealed an interaction effect, post-hoc tests were performed with the Holm-Bonferroni-correction.

	Learning trials				
	Stationary		Mobile		
	**MTLR** (n = 10)	**Control** (n = 20)	**MTLR** (n = 10)	**Control** (n = 20)	
**Spatial memory**					p-value
Memory score in percent	n.a.	n.a.	n.a.	n.a.	n.a.
**Spatial precision**					
Scatter as distance in virtual units	n.a.	n.a.	n.a.	n.a.	n.a.
**Navigation efficiency**					
Latency to final location in seconds	46.7 ± 17.4<br>7.3 – 86.1	19.8 ± 2.3<br>15.1 – 24.5	23.9 ± 4.9<br>12.9 – 34.9	15.4 ± 1.1<br>13.1 – 17.7	G*S: 0.074<br>S: 0.012<br>G: 0.018
Path error to final location in percent	745.5 ± 175.5<br>348.5 – 1142.5	530.5 ± 207.3<br>96.6 – 964.5	226.1 ± 65.1<br>78.8 – 373.3	170.5 ± 21.0<br>126.6 – 214.4	G*S: 0.606<br>S: 0.007<br>G: 0.431
Surface coverage in percent	38.8 ± 3.5<br>30.9 – 46.8	30.2 ± 1.9<br>26.1 – 34.3	24.5 ± 1.8<br>20.4 – 28.6	23.1 ± 1.6<br>19.8 – 26.4	G*S: 0.069<br>S: < 0.001<br>G: 0.039
**Navigation strategies**					
Search accuracy/ avg. distance to final location in virtual units	0.42 ± 0.01 a<br>0.39 – 0.45	0.38 ± 0.01 b<br>0.35 – 0.40	0.34 ± 0.01 c<br>0.31 – 0.37	0.35 ± 0.01 d<br>0.33– 0.38	G*S: 0.040<br>a*b = 0.081<br>a*c = 0.003<br>b*d = 0.163<br>c*d = 0.566
Angular velocity/ idPhi (Integrated over the initial 5 seconds)	0.0060 ± 0.0006<br>0.0046 – 0.0075	0.0076 ± 0.0004<br>0.0067 – 0.0084	0.0081 ± 0.0005<br>0.0070 – 0.0091	0.0105 ± 0.0005<br>0.0093 – 0.0116	G*S: 0.295<br>S: < 0.001<br>G: 0.006
Path replication/ distance between paths in virtual units	n.a.	n.a.	n.a.	n.a.	n.a.

Supplementary table 4

Supplementary table 4. Table of results for influence of session order on experimental variables.

	Learning trials	Probe trials
**Spatial memory**		
Memory score in percent	n.a.	$F_{(1,27)} = 0.048$, $p = 0.828$ $\omega^2 = 0.0$
**Spatial precision**		
Scatter as distance in virtual units	n.a.	$F_{(1,52)} = 0.181$, $p = 0.673$, $\omega^2 = 0.0$
**Navigation efficiency**		
Latency to final location in seconds	$F_{(1,27)} = 0.00$, $p = 0.996$, $\omega^2 = 0.0$	$F_{(1,27)} = 8.096$, $p = 0.008$, $\omega^2 = 0.20$
Path error to final location in percent	$F_{(1,52)} = 0.00$, $p = 0.992$, $\omega^2 = 0.0$	$F_{(1,27)} = 21.206$, $p < 0.001$, $\omega^2 = 0.41$
Surface coverage in percent	$F_{(1,27)} = 1.871$, $p = 0.183$, $\omega^2 = 0.03$	$F_{(1,27)} = 12.404$, $p = 0.002$, $\omega^2 = 0.28$
**Navigation strategies**		
Search accuracy/ avg. distance to final location in virtual units	$F_{(1,52)} = 1.166$, $p = 0.285$, $\omega^2 = 0.00$	$F_{(1,27)} = 1.462$, $p = 0.810$, $\omega^2 = 0.02$
Angular velocity/ idPhi (Integrated over the initial 5 seconds)	$F_{(1,27)} = 0.354$, $p = 0.557$, $\omega^2 = 0.00$	$F_{(1,52)} = 0.427$, $p = 0.517$, $\omega^2 = 0.00$
Path replication/ distance between paths in virtual units	n.a.	$F_{(1,52)} = 0.162$, $p = 0.689$, $\omega^2 = 0.00$

Supplementary methods 2

Electroencephalography

To investigate brain activity during spatial navigation, we equipped all participants with high-density electroencephalography (EEG) with 128 channels synchronized to the virtual or physical motion streams using the lab streaming layer (Kothe, 2014), https://github.com/sccn/labstreaminglayer). EEG data was recorded with a nominal sampling rate of 1000 Hz and band-pass filtered from 0.016 Hz to 500 Hz (BrainAmp Move System, Brain Products, Gilching, Germany). An elastic cap with an equidistant layout (EASYCAP, Herrsching, Germany) was used and the data were referenced to an electrode located closest to the FCz electrode of the extended 10% system. Impedances were kept below 20kΩ and electrode locations were digitized using an optical tracking system (Polaris Vicra, NDI, Waterloo, ON, Canada). For the present study, the EEG data will not be reported. Instead, the analyses focus on the behavioral performance and motion profile of participants.
