# Supplementary material for: Multisensory input modulates memory-guided spatial navigation in humans
Source: Commun Biol. 2023 Nov 14;6:1167. doi: 10.1038/s42003-023-05522-6 (PMC10646091; doi:10.1038/s42003-023-05522-6)
Supplement: Supplementary file 3 — Reporting summary [file 42003_2023_5522_MOESM3_ESM.pdf]

nature portfolio

Corresponding author(s): Deetje Iggena
Last updated by author(s): Oct 13, 2023

# Reporting Summary

Nature Portfolio wishes to improve the reproducibility of the work that we publish. This form provides structure for consistency and transparency in reporting. For further information on Nature Portfolio policies, see our Editorial Policies and the Editorial Policy Checklist.

## Statistics

For all statistical analyses, confirm that the following items are present in the figure legend, table legend, main text, or Methods section.

| n/a | Confirmed | |
|---|---|---|
| ☐ | ☒ | The exact sample size (*n*) for each experimental group/condition, given as a discrete number and unit of measurement |
| ☐ | ☒ | A statement on whether measurements were taken from distinct samples or whether the same sample was measured repeatedly |
| ☐ | ☒ | The statistical test(s) used AND whether they are one- or two-sided<br>*Only common tests should be described solely by name; describe more complex techniques in the Methods section.* |
| ☐ | ☒ | A description of all covariates tested |
| ☐ | ☒ | A description of any assumptions or corrections, such as tests of normality and adjustment for multiple comparisons |
| ☐ | ☒ | A full description of the statistical parameters including central tendency (e.g. means) or other basic estimates (e.g. regression coefficient) AND variation (e.g. standard deviation) or associated estimates of uncertainty (e.g. confidence intervals) |
| ☐ | ☒ | For null hypothesis testing, the test statistic (e.g. $F$, $t$, $r$) with confidence intervals, effect sizes, degrees of freedom and $P$ value noted<br>*Give P values as exact values whenever suitable.* |
| ☒ | ☐ | For Bayesian analysis, information on the choice of priors and Markov chain Monte Carlo settings |
| ☒ | ☐ | For hierarchical and complex designs, identification of the appropriate level for tests and full reporting of outcomes |
| ☐ | ☒ | Estimates of effect sizes (e.g. Cohen's *d*, Pearson's *r*), indicating how they were calculated |

*Our web collection on statistics for biologists contains articles on many of the points above.*

## Software and code

Policy information about availability of computer code

| | |
|---|---|
| Data collection | The virtual water maze was programmed in Unity 3D (v.2018.4.13f1) with C#. The software for the virtual water maze is available upon reasonable request. Please contact the corresponding author. |
| Data analysis | Matlab-functions (MathWorks® Matlab (version 2021a)) and R-function (R (v. 3.5)) are available on Open Science Framework: https://osf.io/u47mj/. |

For manuscripts utilizing custom algorithms or software that are central to the research but not yet described in published literature, software must be made available to editors and reviewers. We strongly encourage code deposition in a community repository (e.g. GitHub). See the Nature Portfolio guidelines for submitting code & software for further information.

## Data

Policy information about availability of data

All manuscripts must include a data availability statement. This statement should provide the following information, where applicable:

- Accession codes, unique identifiers, or web links for publicly available datasets
- A description of any restrictions on data availability
- For clinical datasets or third party data, please ensure that the statement adheres to our policy

Data, matlab- and R-functions is available at the Open Science Framework (osf) at: https://osf.io/u47mj/. Doi 10.17605/OSF.IO/U47MJ)

## Research involving human participants, their data, or biological material

Policy information about studies with human participants or human data. See also policy information about sex, gender (identity/presentation), and sexual orientation and race, ethnicity and racism.

| | |
|---|---|
| Reporting on sex and gender | Sex is given in Table 1 on the participant data: MTLR: 6f/4m; control 12f/8m. Sex and gender were the same for all participants, this was determined by self-report. Sex was a covariate in the linear mixed model. |
| Reporting on race, ethnicity, or other socially relevant groupings | One patient is Asian, all other participants were Caucasian. Assessment was done by researchers. All participants grew up in Germany. We do not expect race or ethnicity to influence memory-guided spatial navigation. |
| Population characteristics | Population characteristics incl. age, sex and education are provided in table 1. |
| Recruitment | Recruitment and inclusion criteria are described in the methods section. Briefly, patients were recruited via Department of Neurology at Charité Universitätsmedizin. Age-, sex- and education matched controls via online advertisment. |
| Ethics oversight | Local ethics committee of Charité-Universitätsmedizin Berlin |

Note that full information on the approval of the study protocol must also be provided in the manuscript.

# Field-specific reporting

Please select the one below that is the best fit for your research. If you are not sure, read the appropriate sections before making your selection.

☐ Life sciences ☒ Behavioural & social sciences ☐ Ecological, evolutionary & environmental sciences

For a reference copy of the document with all sections, see nature.com/documents/nr-reporting-summary-flat.pdf

# Behavioural & social sciences study design

All studies must disclose on these points even when the disclosure is negative.

| | |
|---|---|
| Study description | We tested the influence of multisensory input on memory-guided spatial navigation. This was a hypothesis confirming study. The data collected was quantitative. |
| Research sample | Subjects with hippocampal lesions (n=10) and their healthy age-, sex., and education matched controls (n=20) participated. Population characteristics incl. age, sex and education are provided in table 1. Sex: MRTL (6f/ 4m), CRTL (12f/ 8m); age: MTRL 41 (22 – 61), CTRL 41 (22 – 61); Years of education: MTRL 16 (12 – 20), CTRL 15.5 (12 – 19) |
| Sampling strategy | Subjects with hippocampal lesions were recruited via the Department of Neurology, healthy subjects via online advertisements. The sample size was calculated based on previous studies from our laboratory. Control subjects were doubled to increase power. |
| Data collection | Data collection was conducted at the BeMoBIL-Lab. The virtual environment was programmed in Unity 3D. In the VR session a HTC vive pro system was used. In the stationary session a screen was used to display the virtual environment and a joystick to navigate in the virtual environment. Data was streamed via Lab Streaming Layer. Experimenters were not blinded. |
| Timing | Data collection was conducted between 01/2020 - 03/2020 and 10/2020 - 12/2021. Break in data collection occured due to Covid-19 restrictions |
| Data exclusions | We excluded one patient because we learned after the experiment that she was suffering from a psychiatric illness.Therefore, her two control subjects were also excluded. One control participant was excluded because of cyber sickness. Exclusion criteria were pre-established. |
| Non-participation | All invited participants carried out the experiment. One participant had to quit the VR session due to cyber-sickness. |
| Randomization | Participants were not randomly assigned to groups, as we compared a patient sample with a corresponding healthy sample. |

# Reporting for specific materials, systems and methods

We require information from authors about some types of materials, experimental systems and methods used in many studies. Here, indicate whether each material, system or method listed is relevant to your study. If you are not sure if a list item applies to your research, read the appropriate section before selecting a response.

## Materials & experimental systems

| n/a | Involved in the study |
| --- | --- |
| ☒ | ☐ Antibodies |
| ☒ | ☐ Eukaryotic cell lines |
| ☒ | ☐ Palaeontology and archaeology |
| ☒ | ☐ Animals and other organisms |
| ☒ | ☐ Clinical data |
| ☒ | ☐ Dual use research of concern |
| ☒ | ☐ Plants |

## Methods

| n/a | Involved in the study |
| --- | --- |
| ☒ | ☐ ChIP-seq |
| ☒ | ☐ Flow cytometry |
| ☒ | ☐ MRI-based neuroimaging |

# Plants

| | |
| --- | --- |
| Seed stocks | *Report on the source of all seed stocks or other plant material used. If applicable, state the seed stock centre and catalogue number. If plant specimens were collected from the field, describe the collection location, date and sampling procedures.* |
| Novel plant genotypes | *Describe the methods by which all novel plant genotypes were produced. This includes those generated by transgenic approaches, gene editing, chemical/radiation-based mutagenesis and hybridization. For transgenic lines, describe the transformation method, the number of independent lines analyzed and the generation upon which experiments were performed. For gene-edited lines, describe the editor used, the endogenous sequence targeted for editing, the targeting guide RNA sequence (if applicable) and how the editor was applied.* |
| Authentication | *Describe any authentication procedures for each seed stock used or novel genotype generated. Describe any experiments used to assess the effect of a mutation and, where applicable, how potential secondary effects (e.g. second site T-DNA insertions, mosiacism, off-target gene editing) were examined.* |
